# Improving departmental psychological safety through a medical school-wide initiative

**DOI:** 10.1186/s12909-024-05794-4

**Published:** 2024-07-25

**Authors:** Kirsten A. Porter-Stransky, Karen J. Horneffer-Ginter, Laura D. Bauler, Kristine M. Gibson, Christopher M. Haymaker, Maggie Rothney

**Affiliations:** 1 Department of Biomedical Sciences, University of South Carolina School of Medicine Greenville, 607 Grove Rd, Greenville, SC 29673 USA; 2 Department of Biomedical Sciences, Western Michigan University, Homer Stryker M.D. School of Medicine, Kalamazoo, MI USA; 3 Western Michigan University Homer Stryker M.D. School of Medicine, Kalamazoo, MI USA; 4 Department of Pediatric and Adolescent Medicine, Western Michigan University Homer Stryker M.D. School of Medicine, Kalamazoo, MI USA; 5 Department of Medical Education, Western Michigan University Homer Stryker M.D. School of Medicine, Kalamazoo, MI USA

**Keywords:** Psychological safety, Academic medicine, Leadership, Healthcare teams

## Abstract

**Background:**

Psychological safety is a team-based phenomenon whereby group members are empowered to ask questions, take appropriate risks, admit mistakes, propose novel ideas, and candidly voice concerns. Growing research supports the benefits of psychological safety in healthcare and education for patient safety, learning, and innovation. However, there is a paucity of research on how to create psychological safety, especially within academic medicine. To meet this need, the present study describes and evaluates a multi-year, medical school-wide psychological safety initiative.

**Methods:**

We created, implemented, and assessed a multi-pronged psychological safety initiative including educational training sessions, departmental champions, videos, infographics, and targeted training for medical school leaders. Employees’ perceptions of psychological safety at both the departmental and institutional levels were assessed annually. The impact of educational training sessions was quantified by post-session surveys.

**Results:**

Deidentified employee surveys revealed a statistically significant increase in departmental psychological safety between the first and second annual surveys. Perceived psychological safety remained lower at the institution-wide level than at the departmental level. No significant differences in psychological safety were observed based on gender, position, or employment length. Post-educational training session surveys showed that the sessions significantly increased knowledge of the topic as well as motivation to create a culture of psychological safety within the medical school.

**Conclusions:**

This study establishes an evidence-based method for increasing psychological safety within medical school departments and serves as a template for other health professions schools seeking to promote psychological safety. Training leadership, faculty, and staff is an important first step towards creating a culture of psychological safety for everyone, including trainees.

## Introduction

Academic medicine needs strong teams of employees to handle the volatile, uncertain, complex, and ambiguous challenges of the current times. The COVID-19 pandemic highlighted the need for creativity, out-of-the-box thinking, risk taking, innovation, and teamwork to advance research, treat patients, and educate the next generation of health professionals. Organizational research supports the need for team members to contribute, take reasonable risks, and not fear punishment for occasional mistakes when navigating complex, novel work problems. Together, these factors contribute to psychological safety, which can be defined as “perceptions of the consequences of taking interpersonal risks in a particular context such as a workplace” [1–3]. Psychological safety has been found to correlate with numerous positive outcomes including team performance, learning, willingness to disclose errors and suggest new ideas, along with better quality of healthcare [1, 3–5]. Importantly, psychological safety does not support careless risks that would harm patients, learners, employees, or the company. Rather, a psychologically safe environment encourages individuals to take calculated risks to advance the institution, science, or learning without fear of punishment if they fail. For example, testing innovative hypotheses is the backbone of scientific and medical discoveries; however, many experiments fail to produce the desired results. These “intelligent failures” form the basis for new hypotheses, experiments, and opportunities; they inevitably result when institutions or employees take the calculated risks needed to advance knowledge or the success of a company [6].

In addition to the business sphere, much of the research on psychological safety has focused on healthcare teams [7, 8]. Indeed, the stakes are high for multidisciplinary clinicians to deliver topnotch care. Both medicine and higher education are hierarchical with powerful professional norms which can inhibit psychological safety [9]. Although literature emphasizes a need to promote psychological safety in medicine and higher education [9], there is a paucity of research on how to effectively foster psychological safety. Indeed, Edmonson and Bransby recently wrote, “We believe that the most glaring gap in the literature pertains to how to create psychological safety. Even with the heavy emphasis in the literature on leadership effects on psychological safety, more research on specific interventions leaders can use to build psychological safety in teams or organizations would be valuable.” [1] To contribute to filling this gap, we designed and implemented a medical school-wide initiative to promote psychological safety, with a focus on training faculty and staff. Although psychological safety is also important for students and residents, we purposefully chose to educate employees first to ensure that inclusive behaviors would be role modeled by faculty and staff and that a psychologically safe learning environment would be established.

This project is based in the theoretical framework that psychological safety is a team-based phenomenon that can be examined on multiple levels including the individual, group, and organizational levels [3, 10]. The majority of psychological safety research has focused on the work-unit level [10]. Relatively fewer studies have examined psychological safety at the organizational level or at multiple timepoints [11–14]. To assess the impact of our multi-faceted psychological safety initiative for academic medicine, we conducted a study using deidentified annual surveys that assessed employees’ perceived psychological safety within their department as well as in the institution. In addition, we conducted post-educational event assessments to evaluate participants’ psychological safety knowledge and motivation resulting from the initiative’s trainings.

## Methods

### ***Setting and participants***

All employees of a private, Midwestern medical school in the United States were invited to participate in the psychological safety initiative. This study was reviewed by the Western Michigan University Homer Stryker M.D. School of Medicine (WMed) Institutional Review Board and deemed exempt.

### ***Psychological safety initiative***

**Fig. 1:**
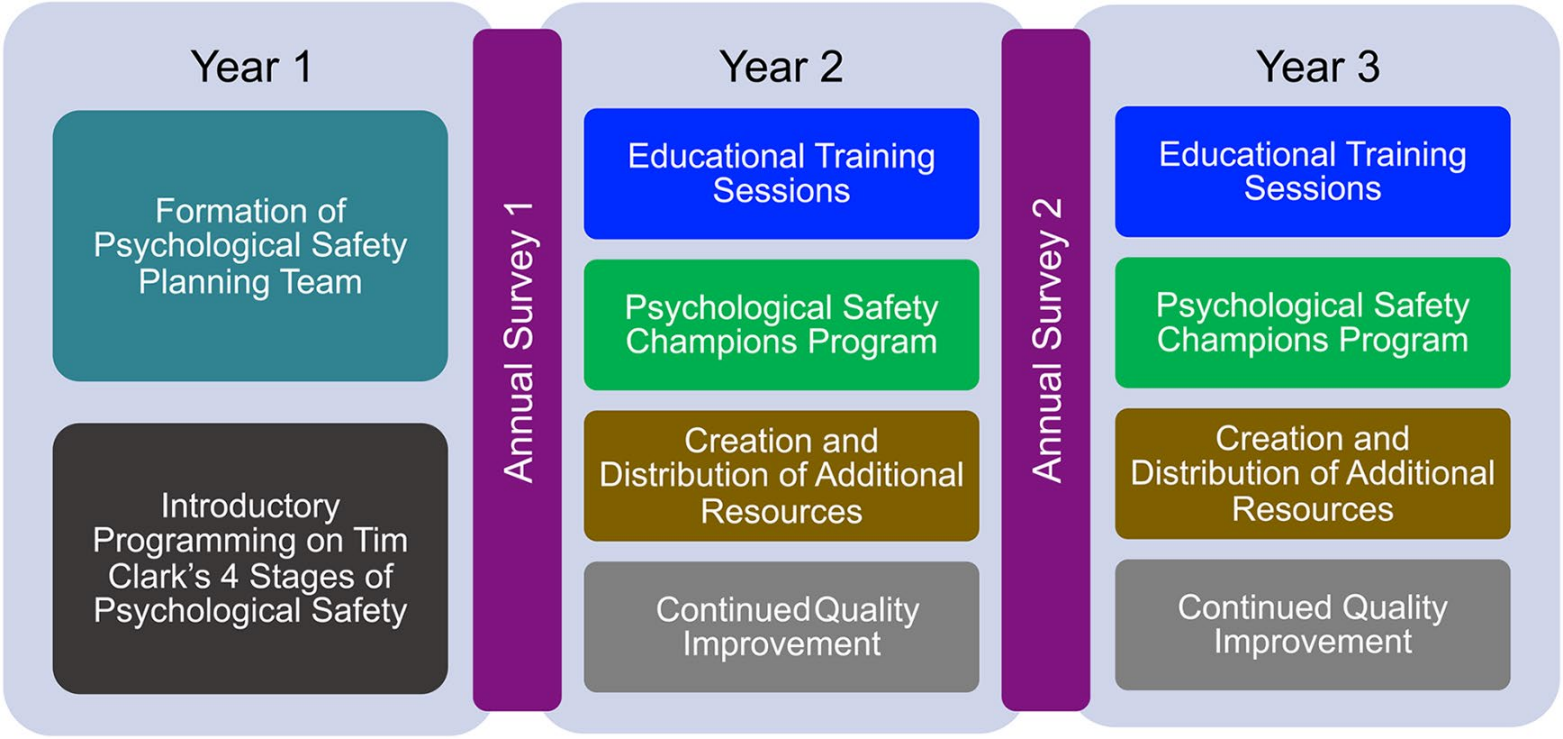
The medical school-wide psychological safety initiative timeline

Figure 1 summarizes the timeframe of our multi-faceted initiative. During the first year, the planning team was formed, and introductory sessions modeled after Tim Clark’s 4-stage model of psychological safety [15] were presented to anyone interested at the university. According to this model, the 4 stages of psychological safety within the workplace include: (1) inclusion safety (allowing others to feel included and accepted), (2) learner safety (allowing others to feel safe and motivated to learn), (3) contributor safety (encouraging others to feel safe enough to contribute and make a difference), and (4) challenger safety (allowing others to feel safe enough to challenge the status quo, innovate, and make things better) [15]. These sessions were active learning sessions that included an overview of each of the 4 stages, breakout groups to brainstorm barriers to each of these stages specific to various roles and departments, in addition to potential solutions to those barriers. During the second and third years, educational training sessions on psychological safety with post-session surveys were rolled out across the medical school. These educational training sessions included an introductory overview of the 4 stages, in addition to topics such as (1) providing and receiving feedback, (2) ensuring a sense of inclusion among team members, and (3) identifying next steps and resources for increasing departmental psychological safety. The psychological safety champions program consists of at least one representative from participating departments who serve to disseminate information about the initiative with their coworkers and encourage departmental participation. The psychological safety planning team created and distributed additional resources including (1) infographics, (2) gamification tools (for each stage of Tim Clark’s model), (3) a brief introductory video, and (4) a departmental psychological safety assessment tool. Readers may contact the second author for additional details and examples of programmatic content. The first annual survey was distributed to all medical school employees after the preliminary year of project planning and introductory sessions. The second survey was distributed at the end of year 2, after the launch of multiple educational training sessions, the psychological safety champions program, and the initial distribution of the various resources. We continue to improve the program and generate additional resources and training opportunities for all employees.

### ***Data collection***

All employees were invited to complete an annual REDCap survey in January 2022 and 2023 (Fig. 1) that included demographic questions and the previously validated Psychological Safety Scale [2, 16] to measure perceived psychological safety. Questions were assessed on a 7-point scale (strongly disagree, disagree, somewhat disagree, neither agree nor disagree, somewhat agree, agree, strongly agree). To assess psychological safety at the team level, questions included “department/unit” (for example, “If you make a mistake in my department/unit, it is often held against you,” which is reverse scored). Similarly to Baer and Frese [11], we changed “department/unit” to our organization’s name to assess psychological safety at the institutional level (for example, “If you make a mistake at WMed, it is often held against you”). All employed faculty and staff received an email containing a REDCap survey link with a unique number, which facilitated pairing the data across years for within-subject analyses.

Following educational training sessions, attendees were sent an optional survey in REDCap. Data were de-identified by an honest broker who sent only the first post-educational assessment from each employee that attended. These surveys utilized a retrospective pre-post methodology [17]. Survey questions probed participants’ psychological safety knowledge and motivation to both learn more and engage in creating psychological safety. Finally, open-ended questions allowed participants to provide comments about next steps or obstacles for increasing psychological safety.

### ***Data analysis***

Quantitative data were analyzed and graphed in GraphPad Prism version 10. Psychological Safety Scale scores were calculated as percentage of maximum scores to improve interpretability for the reader and facilitate comparisons with future studies. Between-subjects ANOVAs were conducted to test whether psychological safety at the departmental or institutional level varied by length of employment at or role within the medical school. A repeated-measures ANOVA with planned contrasts was used to examine differences in psychological safety between timepoints and between departmental and institutional levels. On the post-educational surveys, responses to the 3 knowledge-based questions were averaged into a knowledge score and participants self-reported ratings of their knowledge before and after the event were compared with a paired t-test. Descriptive statistics were used to report participants’ self-reported motivation for and optimism about creating a culture of psychological safety.

We performed a thematic analysis [18] on the responses to optional, open-ended questions as an adjunct analysis [19]. The two questions were, “What next steps would you like to see happen at WMed to increase psychological safety?” and “What do you see as the biggest obstacles to creating more psychological safety at WMed?” First, all authors of this manuscript read respondents’ deidentified comments individually. Then, we met to discuss the qualitative data and themes that we identified. After this discussion, the first author coded the comments based upon identified themes and shared the results with the other authors to verify. Primarily, insights were used internally to improve the psychological safety initiative. In this paper, we share broad qualitative results that would be applicable to other medical schools’ psychological safety initiatives.

## Results

### ***Assessing psychological safety***

A total of 97 employees completed the annual psychological safety surveys for two consecutive years. The sample size represents 14.4% of all employees (674) at the time of Annual Survey 1. The demographics of study participants largely mirrored that of the medical school’s employed population (Table 1).

**Table 1:** Demographics of study participants. *N* = 97 participants completed both annual surveys. *N* = 67 participants completed post-educational surveys. The participant pools were representative of the employed population (674 and 783 employees at the times of the surveys 1 and 2, respectively) at the medical school. Clinical faculty and staff were defined as having patient care responsibilities. Non-clinical faculty included teaching, research, and/or administratively-focused faculty without patient care duties

	Participants in annual surveys	Employed population at time of Annual Survey 1	Employed population at time of Annual Survey 2	Participants in post-educational surveys
**Position**				
Non-clinical faculty	15 (15.5%)	58 (8.6%)	62 (7.9%)	12 (17.9%)
Clinical faculty	22 (22.7%)	102 (15.1%)	110 (14.0%)	15 (22.4%)
Non-clinical staff	50 (51.5%)	387 (57.4%)	457 (58.4%)	34 (50.7%)
Clinical staff	9 (9.3%)	127 (18.8%)	154 (19.7%)	4 (6.0%)
No response	1 (1.0%)			2 (3.0%)
**Gender**				
Male	22 (22.7%)	221 (32.8%)	269 (34.4%)	17 (25.4%)
Female	74 (76.3%)	453 (67.2%)	500 (63.9%)	48 (71.6%)
Non-binary or no response	1 (1.0%)	0 (0.0%)	14 (1.8%)	2 (3.0%)
**Length of employment w/ institution**	Length at time of 2nd survey			
0 to < 1 year	--			8 (11.9%)
1 to < 2 years	9 (9.3%)			7 (10.4%)
2 to < 4 years	20 (20.6%)			14 (20.9%)
4 to < 6 years	15 (15.5%)			7 (10.4%)
6 to 10 years	30 (30.9%)			19 (28.4%)
>10 years	21 (21.6%)			9 (13.4%)
No response	2 (2.1%)			3 (4.5%)

**Fig. 2:**
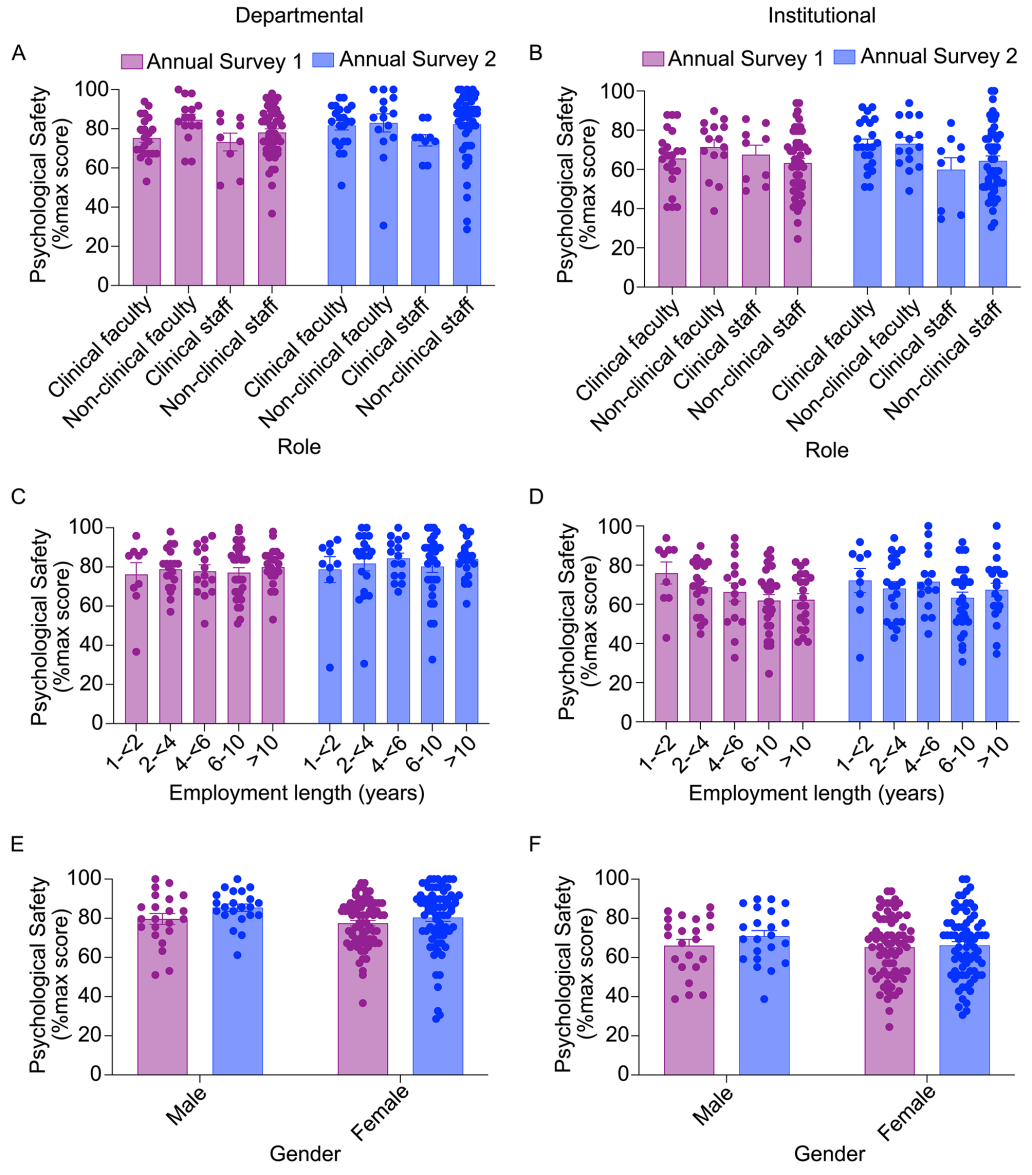
Psychological safety by demographics. **A-B)** At the departmental **(A)** or institutional **(B)** level, psychological safety did not significantly differ based on role within the medical school. **C-D)** Psychological safety scores also did not differ at the departmental **(C)** or institutional **(D)** level based upon employment length. **E-F)** Similarly, psychological safety at the departmental **(E)** or institutional **(F)** level did not significantly differ based upon gender. *N* = 95–96, error bars = SEM.

Due to the hierarchical natures of academia and medicine, it is possible that psychological safety varies by role or length of employment. However, we did not observe any statistically significant differences by role for departmental psychological safety (Fig. 2A; main effect of role, *F*_(3, 92)_ = 1.55, *p* = 0.206) or institutional psychological safety (Fig. 2B; main effect of role, *F*_(3, 92)_ = 1.924, *p* = 0.131). Similarly, we did not detect statistically significant differences by length of employment for departmental psychological safety (Fig. 2C; main effect of employment length, *F*_(4, 90)_ = 0.400, *p* = 0.808) or institutional psychological safety (Fig. 2D; main effect of employment length, *F*_(4, 90)_ = 1.549, *p* = 0.195). We also examined whether psychological safety varied between males and females and detected no statistically significant differences at the departmental (Fig. 2E; main effect of gender, *F*_(1, 94)_ = 1.648, *p* = 0.202) or institutional (Fig. 2F; main effect of gender, *F*_(1, 94)_ = 0.677, *p* = 0.413) level. Because there were not robust differences in psychological safety by role, employment length, or gender, subsequent analyses grouped all participants together.

**Fig. 3:**
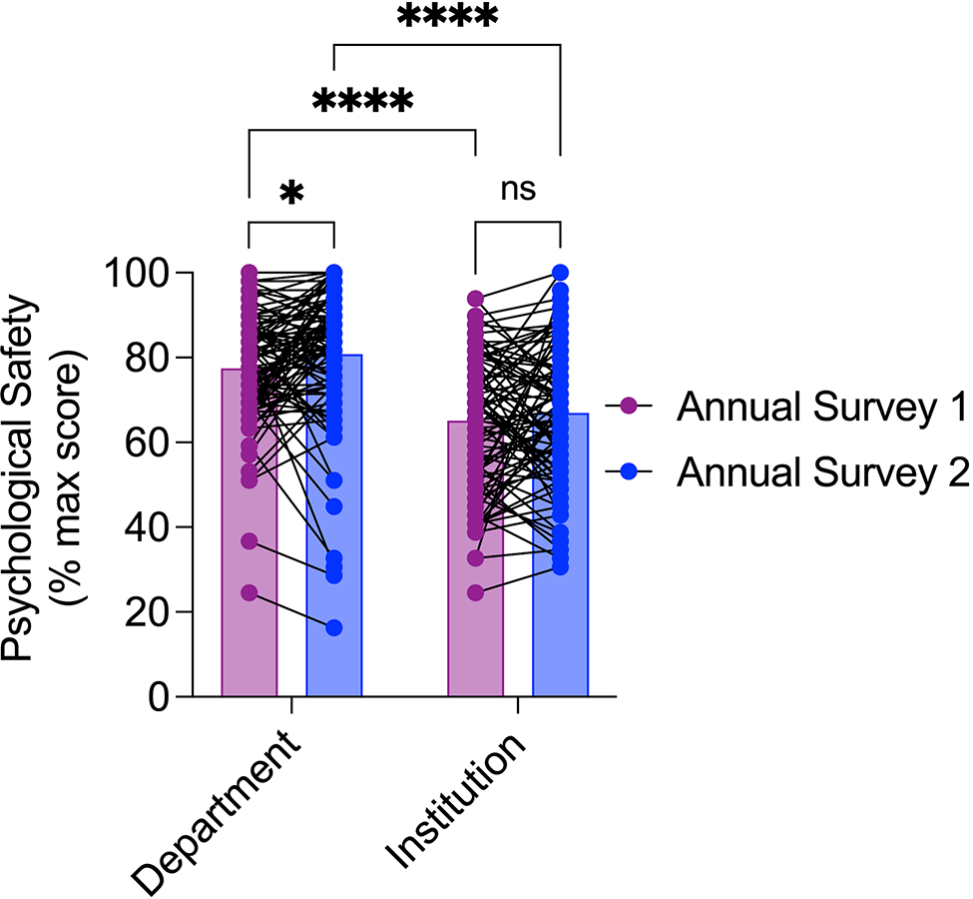
Annual psychological safety scores at the departmental and institutional levels. Psychological safety at the departmental level increased during the initiative and was consistently higher than at the intuition-wide level. *N* = 97, *p < 0.05, ****p < 0.0001, ns = not statistically significant

Consistently across both years, participants rated higher psychological safety within their department than within the institution at large (Fig. 3; main effect, *F*_(1, 96)_ = 87.71, *p* < 0.0001; contrasts, *p* < 0.0001 for both years). Following implementation of the psychological safety initiative, a statistically significant increase in psychological safety was observed (Fig. 3; main effect, *F*_(1, 96)_ = 4.00, *p* = 0.049). Contrasts revealed an increase in departmental psychological safety (*p* = 0.027) but not in institution-wide psychological safety (*p* = 0.259) between the two annual surveys (Fig. 3).

### ***Assessing educational training sessions***

**Fig. 4:**
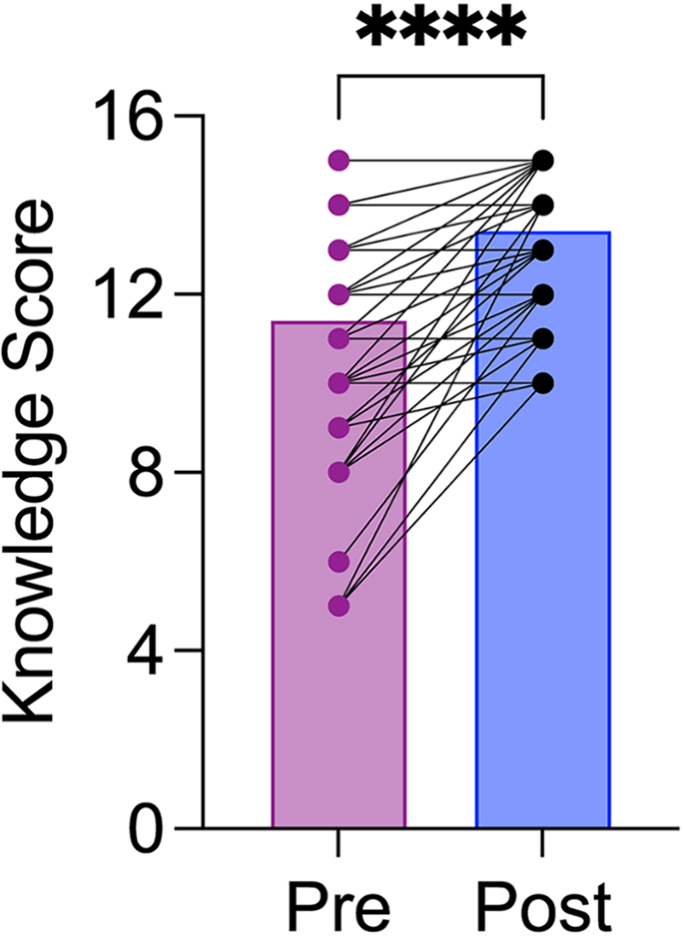
Increases in psychological safety knowledge following educational training sessions. *N* = 67, ****p < 0.0001

A total of 67 of the 136 participants who attended educational training sessions completed post-educational surveys, representing a 49% response rate. In terms of gender, employment type, and length of employment, the employees who participated in the educational training sessions were comparable to those who completed the psychological safety surveys (Table 1). Following the sessions, there was a statistically significant increase in participant knowledge about psychological safety (Fig. 4; *t*_(66)_ = 7.775, *p* < 0.0001). Additionally, at the end of the training events, 88% of participants were motivated to learn more about psychological safety, 95% were motivated to create a culture of psychological safety within the institution, and 89% were hopeful that we can create a culture of psychological safety.

Responses to the open-ended prompts on the post-session surveys were used internally for continuous quality improvement of the initiative. Of participants that responded to these questions, 61% included content regarding leadership. Specifically, most of these comments captured the hierarchical nature of academic medicine and/or the importance of training leaders to model psychological safety from the top down. If leaders fail to foster a psychologically safe work climate, employees and trainees will not feel psychologically safe. Using this feedback, the team developed educational training sessions specifically for higher leadership within the institution. Now, all senior leaders and managers have received at least introductory-level training in how to foster psychological safety in their interactions with others, and a psychological safety module has been added to our institution’s ongoing leadership training series.

## Discussion

The present study begins filling what was termed a glaring gap in the literature [1] – how to create psychological safety – by delineating and evaluating one such programmatic effort. While prior research has studied psychological safety within health care or education [9], the present study provides a template for educating an academic medicine organization, including teaching, research, and healthcare faculty and staff, about psychological safety. Furthermore, this study contributes to the literature by comparing employees’ perceived psychological safety within their work unit versus the larger institution, which is important, yet rarely done.

Research has shown that although most surveyed executives report that improving workplace culture is a necessary priority, most corporate cultural changes are slow and challenging [20]. Although culture changes can take years, we observed a statistically significant increase in departmental psychological safety within one year’s timeframe. This encouraging finding supports the effectiveness of our psychological safety initiative. It may, however, take longer to improve feelings of psychological safety within the organization at large. In multi-faceted organizations, consistent engagement among employees of all departments is challenging. Additionally, many employed faculty and staff have fewer direct interactions with higher leadership than with their departmental supervisors. Regular opportunities for such interactions can be necessary to generate institutional-wide cultural norms and psychological safety. Indeed, Newman et al. suggest, “psychological safety is likely to be more potent and meaningful at the team level, rather than the organizational level.” [10] Focusing on improving psychological safety on the team level may be the catalyst for further institution-wide psychological safety. Such efforts are consistent with findings which suggest that 70% of what influences employees’ feelings about their workplace culture can be attributed to the team (vs. organization-wide) level [21]. Further research on how an organization’s size impacts psychological safety and differences between departmental and institutional psychological safety is warranted. Additionally, although we did not observe differences in psychological safety based on gender or time in role, future research should continue to investigate potential influences of gender, seniority, and race on psychological safety [22, 23].

Our longitudinal study demonstrates that psychological safety can be improved at the departmental level. We hypothesize that culture change may progress from the departmental to institution-wide level, but how long such transformations may take has yet to be determined within a medical school environment. Additional strengths of this study include using the established Psychological Safety Scale [2], examining psychological safety at both the departmental and institutional levels [1], and examining changes in psychological safety across time [14]. While we sought to improve psychological safety at both the departmental and institutional levels, the fact that we observed an increase at one level (departmental) but not the other (institutional) demonstrates that scores did not increase merely as an effect of the passage of time or retaking the same survey. These findings support the methodological validity and reliability of the results. Furthermore, culture change is an inherently slow process among healthcare organizations that requires building trust and increasing communication among individuals, but it is worth the time, leading to improvements in education, employee satisfaction, and patient care [24].

Accumulating research emphasizes the importance of supportive leadership behaviors to model and cultivate psychological safety [10]. Consistent with prior work, our participants identified the importance of institutional leaders and supervisors modeling psychological safety from the top down. Leadership sets a tone for how the organization functions, and medical schools often have a culture of fear [25]. Trust, both of the leader and from the leader, at each level within an organization, substantially impacts how psychologically safe individuals feel within an institution [25, 26]. Although trust was not specifically measured within the present study, it is arguably the essence of psychological safety. Trust dictates how vulnerable someone is willing to be, and therefore how psychologically safe someone feels: can they ask questions, can they admit not knowing, can they take risks, and can they challenge the status quo without fear of consequences? Without trust among employees, across departments, and with leadership, psychological safety is unachievable and leads to reduced communication, decreased productivity, and increased employee turnover [27]. Furthermore, as an organization increases in size, an individual’s ability to know, communicate effectively with, and trust colleagues diminishes; thus, there is an inverse relationship between team size and team performance that is mediated by psychological safety [28]. Our qualitative finding of the importance of leadership reinforced our commitment to training those in leadership roles. Although we plan to expand training to medical students and residents, we emphasize the importance of starting with leaders and employees.

This study has several limitations. First, it was conducted at one medical school; the scholarly field would benefit from additional research at other health professions schools. However, such initiatives may be most effective when tailored to the institution’s employees and culture, limiting the practicality of applying uniform curricula across different institutions. Second, introductory programming about psychological safety began before the first annual survey; therefore, data from the first survey do not reflect a true baseline. Although the initial sessions may have begun impacting participants’ experiences of and views on psychological safety, they did not obscure detecting an improvement in departmental psychological safety between the two annual surveys. Third, to preserve participants’ confidentiality, we did not collect names of participants’ departments or specific work units. Although we observed a significant increase in departmental psychological safety, there could be differences across departments or units that warrant further study. Indeed, prior research has found differences in psychological safety among various work units within an organization [2, 29]. Our organization’s psychological safety team is offering optional consultations for departments that are prioritizing further advancement in this domain. Fourth, although the demographics of study participants were generally representative of the employed population at the medical school (Table 1), the sample size for those completing the psychological safety surveys was relatively small (14.4% of study-eligible employees completed both annual surveys). Fifth, we have yet to observe a statistically significant increase in psychological safety at the institutional level. As mentioned above, this could be due to the time it takes to effect widespread cultural change. Alternatively, it could reflect either employees’ lack of connection with higher leadership or a limited impact of our initiative. By their nature, multi-faceted organizations, such as medical schools, tend to generate greater connection within one’s department than to the organization as a whole. Therefore, such initiatives simply may be more effective at the departmental versus institutional level. It may also be that psychological safety is primarily a team-based phenomenon rather than a large institution-wide phenomenon. Our finding might also reflect what will come to be seen as a realistic progression in which changes in psychological safety are first seen at the departmental level.

In conclusion, the present study serves as a novel evidence-based method to improve employee’s psychological safety within departments throughout a medical school. Future research should continue analyzing the promotion of psychological safety at both the departmental and institution-wide levels.

## Data Availability

The datasets generated and analyzed during the current study are not publicly available to preserve participants’ confidentiality but are available from the corresponding author on reasonable request.
